# SHORT EDUCATION INTERVENTION MITIGATES AGEISM IN EARLY ADOLESCENCE

**DOI:** 10.1093/geroni/igae098.0186

**Published:** 2024-12-31

**Authors:** Assaf Suberry, Sarit Okun, Liat Ayalon

**Affiliations:** School of Social Work, Bar-Ilan University, Saad, HaDarom, Israel; The Impact Center for Study Ageism and Old Age, Ramat Gan, HaMerkaz, Israel; Bar Ilan University, Bar Ilan University, HaMerkaz, Israel

## Abstract

This study explored the effectiveness of a 90-minute workshop in reducing ageism and fostering social activism among 318 Israeli teenagers (11-15 years old, 73.4% female). The workshop aimed to provide accurate information, counter-stereotypes, and peer discussion through activities like talks, games, videos, and meme creation to promote a more inclusive society. The intervention significantly improved familiarity with the concept of ageism, with teenagers drawing parallels to discrimination and racism. The Children’s Attitudes Towards Elderly (CATE; Seefeldt et al., 1977) was administered before and after the intervention. A dependent samples t-test indicated a significant mean difference in age stereotypes within the same participants, with a medium effect size, (t(317)=-11.91, p<.001, Cohen’s d=.51). Building on prior findings, this study adds to the evidence that educational interventions can improve attitudes towards older adults. Interestingly, while a wider range of age stereotypes emerged post-workshop, a shift towards positive perceptions was observed, particularly among females. Importantly, two-thirds of the teenagers’ created memes targeted ageism against older adults, highlighting its prevalence. However, nearly 20% promoted age-inclusivity, and 17% addressed ageism towards younger generations. These findings suggest the intervention’s potential in fostering social change and highlight the need for further exploration of ageism in youth populations.

